# StereoTactic radiotherapy for wet Age-Related macular degeneration (STAR): study protocol for a randomised controlled clinical trial

**DOI:** 10.1186/s13063-016-1676-7

**Published:** 2016-11-24

**Authors:** James E. Neffendorf, Riti Desai, Yanzhong Wang, Joanna Kelly, Caroline Murphy, Barnaby C. Reeves, Usha Chakravarthy, Sarah Wordsworth, Cornelius Lewis, Janet Peacock, Shahir Uddin, Joe M. O’Sullivan, Timothy L. Jackson

**Affiliations:** 1 Department of Ophthalmology, King’s College Hospital, London, UK; 2 School of Medicine, King’s College London, London, UK; 3 Division of Health and Social Care Research, King’s College London, London, UK; 4 King’s Clinical Trials Unit, King’s College London, London, UK; 5 School of Clinical Sciences, University of Bristol, Bristol, UK; 6 Central Angiographic Resource Facility, Queen’s University of Belfast, Belfast, UK; 7 Health Economics Research Centre, University of Oxford, Oxford, UK; 8 Medical Physics and Engineering, King’s College Hospital, London, UK; 9 Centre for Cancer Research and Cell Biology, Queen’s University of Belfast, Belfast, UK

**Keywords:** Anti-vascular endothelial growth factor, VEGF, Neovascular age-related macular degeneration, Wet age-related macular degeneration, Radiation, Ranibizumab, STAR study, Stereotactic radiotherapy

## Abstract

**Background:**

The standard of care for neovascular age-related macular degeneration (nAMD) involves ongoing intravitreal injections of anti-angiogenic drugs targeting vascular endothelial growth factor (VEGF). The most commonly used anti-VEGF drugs are ranibizumab, bevacizumab and aflibercept. The main objective of the STAR trial is to determine if stereotactic radiotherapy can reduce the number of anti-VEGF injections that patients with nAMD require.

**Methods/design:**

STAR is a multicentre, double-masked, randomised, sham-controlled clinical trial. It evaluates a new device (manufactured by Oraya, Newark, CA, USA) designed to deliver stereotactic radiotherapy (SRT) to nAMD lesions. The trial enrols participants with chronic, active nAMD. Participants receive a single SRT treatment (16 Gy or sham) with a concomitant baseline intravitreal injection of 0.5 mg ranibizumab. Thereafter, they attend every month for 24 months, and ranibizumab is administered at the visit if retreatment criteria are met. The primary outcome is the number of *pro re nata* ranibizumab injections during the first 24 months. Secondary outcomes include visual acuity, lesion morphology, quality of life and safety. Additional visits occur at 36 and 48 months to inspect for radiation retinopathy.

The target sample size of 411 participants (randomised 2:1 in favour of radiation) is designed to detect a reduction of 2.5 injections against ranibizumab monotherapy, at 90% power, and a significance level (alpha) of 0.025 (one-sided two-sample *t* test). This gives 97% power to detect non-inferiority of visual acuity at a five-letter margin. The primary analyses will be by intention to treat.

**Discussion:**

The safety and efficacy outcomes will help determine the role of SRT in the management of chronic, active nAMD.

**Trial registration:**

International Standard Randomised Controlled Trial Number: ISRCTN12884465. Registered on 28 November 2014.

ClinicalTrials.gov: NCT02243878. Registered on 17 September 2014.

## Background

Age-related macular degeneration (AMD) is the leading cause of loss of vision in the elderly in developed countries [1]. There are two forms of AMD: a ‘dry’ atrophic form and a ‘wet’ neovascular form. Wet AMD is associated with the formation of choroidal neovascularisation (CNV), which leaks blood and fluid into and under the macula, causing macular scarring and central vision loss. The overall prevalence of wet AMD is estimated to be 1.2%, increasing to 2.5% in those aged 65 or older and to 6.3% in those aged 80 years or older [2]. As the population ages, the prevalence is projected to increase by one-third over 8 years [2]. The standard of care for wet AMD involves intravitreal injection of drugs targeting vascular endothelial growth factor (VEGF), most commonly bevacizumab, ranibizumab and aflibercept.

Ranibizumab (Lucentis®, Novartis, Frimley, UK), a monoclonal fragment derived from the anti-VEGF antibody bevacizumab, was approved by the US Food and Drug Administration in June 2006 for the treatment of wet AMD. The Anti-VEGF antibody for the Treatment of Predominantly Classic Choroidal Neovascularisation in AMD (ANCHOR) study found that 96% of ranibizumab-treated patients maintained or improved vision compared with 64% of patients treated with photodynamic therapy [3]. The Minimally Classic/Occult Trial of the Anti-VEGF Antibody Ranibizumab in the Treatment of Neovascular Age-Related Macular Degeneration (MARINA) study demonstrated that 95% of ranibizumab-treated patients experienced visual improvement or stabilisation compared with 62% of sham-treated patients after 12 months [4]. Moreover, 34% of patients experienced 15 letter increases in vision. In both the MARINA and ANCHOR studies, patients received monthly ranibizumab injections [3, 4]. Current standard ranibizumab treatment commences with monthly injections for 3 months, typically followed by treatment on an as-needed (*pro re nata*, *prn*) basis if there is evidence of disease activity.

Whilst generally safe and effective, anti-VEGF monotherapy entails a considerable burden of care for most patients with neovascular AMD (nAMD), with regular hospital review for the remainder of their life and repeated intraocular injections. Further, not all patients respond fully, and some of those who do, fail to maintain their response over time [5]. There is therefore an unmet need for a more durable treatment that reduces the economic cost of nAMD treatment and the considerable burden faced by patients who require chronic anti-VEGF monotherapy.

Theoretical, experimental and clinical evidence suggests that low-dose external beam radiation is a useful therapy in nAMD. Radiation has several potential benefits. First, it is known to attenuate the inflammatory response and is therefore likely to attenuate the acute and delayed inflammatory response that is thought to play a role in CNV reactivation [6]. Second, radiation inhibits fibroblasts and thus reduces scar formation, e.g. in its use for dermal keloids [7]. Scarring is a key contributor to vision loss in nAMD. Third, radiation leads to the death of rapidly dividing endothelial cells — the main pathological component of CNV complexes [8].

The StereoTactic radiotherapy for wet Age-Related macular degeneration (STAR) trial investigates a new CE marked device, manufactured by Oraya (Newark, CA, USA), that uses radiation to treat nAMD, in a process called stereotactic radiotherapy (SRT) [9–11]. Oraya’s SRT system is an outpatient-based radiotherapy platform that provides stereotactic application of low-energy X-ray radiation to the retina [12–14]. The system uses three highly collimated beams of radiation that pass through the inferior sclera to overlap at the macula, administered in a single treatment session [15]. It uses a contact lens system to hold the eye in the correct position for radiotherapy delivery, with eye tracking software. The SRT device delivers radiation over a 4-mm treatment zone which receives at least 90% of the intended dose [12, 13].

After favourable phase I data, the IRay plus Anti-VEGF Treatment For Patients with Wet AMD (INTREPID) study was initiated to further investigate SRT. This phase II, randomised, double-masked, sham-controlled, dose-ranging (16 and 24 Gy arms) trial recruited 230 patients. It found that a single dose of SRT significantly reduces intravitreal injections required over 1 and 2 years [16, 17]. In terms of safety, SRT was shown to induce microvascular abnormalities, but in only 1% of eyes was vision possibly affected at the 2-year follow-up. A subgroup analysis showed the best responders were those where the AMD lesion had a greatest linear dimension <4 mm (corresponding to the 90% isodose size) and when the lesion was actively leaking at the time of SRT [18].

The STAR trial is a phase III study that builds on the phase II INTREPID study, targeting patients with chronic active nAMD, but it selectively recruits those thought most responsive to SRT, namely those with active leakage at enrolment and with lesions <4 mm [18]. It aims to determine if SRT with *prn* ranibizumab is a safe and effective treatment compared to *prn* ranibizumab monotherapy.

## Methods/design

### Overview

This phase III, double-masked, randomised controlled study will randomise 411 participants to receive either 16-Gy or sham SRT in a 2:1 allocation (favouring 16 Gy), with a concomitant baseline intravitreal injection of 0.5 mg ranibizumab. Thereafter, participants will attend clinic for a review every month (28 days) for 24 months, and ranibizumab will be administered at the visit if defined retreatment criteria are met (termed ranibizumab monthly *prn*). Two safety visits occur subsequently, one at 36 months and the other at 48 months.

Ethical approval was granted by the National Health Service (NHS) Health Research Authority National Research Ethics Service (NRES) Committee London — City and East on 23 October 2013 (REC reference: 13/LO/1207, IRAS project ID: 86810).

**Fig. 1:**
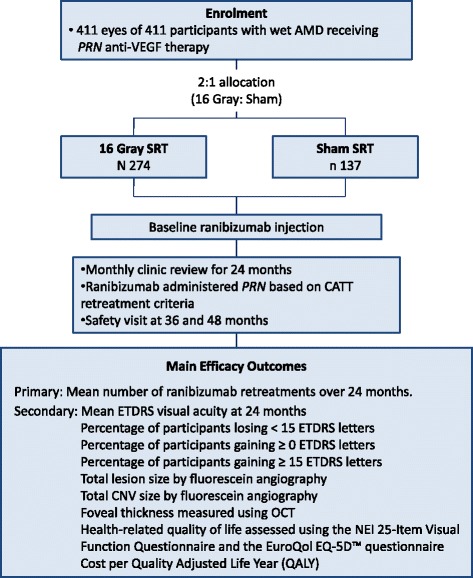
Summary of trial design

The trial is summarised in Fig. 1.

### Eligibility

#### Inclusion criteria

The inclusion criteria are as follows:

- Participants must have neovascular AMD in the study eye, for which they have received at least three prior intravitreal injections of either bevacizumab (Avastin), aflibercept (Eylea), ranibizumab (Lucentis) or pegaptanib (Macugen).
- Participants must have received an anti-VEGF injection in the study eye within 4 months prior to enrolment.
- Participants must require treatment with anti-VEGF therapy at the time of enrolment due to optical coherence tomography (OCT) scan evidence of subretinal fluid and/or cystoid macular oedema, and have a macular volume that is greater than the 95^th^ percentile of normal for the spectral domain (SD)-OCT machines used in the investigational sites.
- Participants must be at least 50 years of age.

#### Exclusion criteria

The exclusion criteria are as follows:

- Disciform scarring that involves the fovea, in the study eye
- Visual acuity (VA) worse than 6/96 (24 Early Treatment Diabetic Retinopathy Study [ETDRS] letters) in the study eye
- Lesion size greater than 4 mm in greatest linear dimension or greater than 2 mm from the centre of the fovea to the furthest point on the lesion perimeter, to include active choroidal neovascular leakage, pigment epithelial detachment and haemorrhage, as determined by fluorescein angiography
- An axial length of less than 20 mm or greater than 26 mm, in the study eye
- Contraindication or sensitivity to contact lens application, including recurrent corneal erosions, in the study eye
- Type 1 or type 2 diabetes mellitus
- Retinopathy in the study eye
- Prior, current or anticipated treatment in the study eye for AMD, other than anti-VEGF agents, including submacular surgery, subfoveal thermal laser photocoagulation, photodynamic therapy (PDT) or transpupillary thermotherapy (TTT)
- Presence of an intravitreal device in the study eye
- Previous radiation therapy to the study eye, head or neck with the exception of radio-iodine treatment for hyperthyroidism, epimacular brachytherapy to the non-study eye or Oraya SRT to the non-study eye
- Inadequate pupillary dilation or significant media opacities in the study eye, including cataracts, which may interfere with visual acuity testing, the clinical evaluation of the posterior segment or fundus imaging
- Study eyes with CNV due to causes other than AMD, including presumed ocular histoplasmosis syndrome (POH), angioid streaks, multifocal choroiditis, choroidal rupture and pathological myopia (greater than 8 dioptres spherical equivalent). Participants with retinal angiomatous proliferation (RAP) or idiopathic polypoidal choroidal vasculopathy (IPCV) are *not* excluded
- Known allergy to intravenous fluorescein, indocyanine green or intravitreal ranibizumab
- Intraocular surgery or laser-assisted in situ keratomileusis (LASIK) in the study eye within 12 weeks prior to enrolment
- Prior pars plana vitrectomy in the study eye
- Current participation in another interventional clinical trial or participation in such a clinical trial within the last 6 months
- Unwilling, unable or unlikely to return for scheduled follow-up for the duration of the trial
- Women who are pregnant at the time of radiotherapy
- Participants with an implantable cardioverter defibrillator (ICD) or pacemaker implant (or any implanted device) where the device labelling specifically contraindicates patients undergoing X-ray radiation
- Any other condition which, in the judgement of the investigator, would prevent the participant from granting informed consent or completing the study, such as dementia and mental illness (including generalised anxiety disorder and claustrophobia)

### Randomisation

Once baseline assessments are complete and consent has been obtained by trial-certified medical staff, participants will be randomised to SRT and sham in a 2:1 ratio. Randomisation is at the patient level and is performed using an online randomisation system set up by the King’s Clinical Trials Unit (KCTU) at King’s College London. Randomisation is stratified by national treatment centre with variable block sizes to ensure that patients are allocated to the two arms within each treatment centre in a 2:1 ratio. The procedure is as follows: The patient travels from his/her local recruiting site, having been determined as eligible. Staff members at the national treatment centre then use the online randomisation system to get an alphanumeric code. This is entered into the Oraya machine, which will then administer sham treatment or active treatment. The person delivering the radiation/sham treatment does not know which has been selected, as the machine fires up and prepares a dose map in the same way for each treatment.

### Outcome measures

#### Primary

The primary outcome will be the number of *prn* ranibizumab injections during the first 24 months of the study.

#### Secondary (at 24 months)

Secondary outcome measures are the following:

- Mean ETDRS VA
- Percentage of participants losing <15 ETDRS letters
- Percentage of participants gaining ≥0 ETDRS letters
- Percentage of participants gaining ≥15 ETDRS letters
- Total lesion size by fluorescein angiography
- Total CNV size by fluorescein angiography
- Foveal thickness measured using OCT
- Health-related quality of life assessed using the National Eye Institute 25-Item Visual Function Questionnaire (VFQ-25) and the EuroQol EQ-5D^TM^ questionnaire
- Cost per quality-adjusted life year (QALY)

### Patient recruitment and consent procedure

Potential participants will be identified from retinal clinics at the trial sites and provided with a Research Ethics Committee (REC)-approved Patient Information Sheet. After at least 24 hours, usually longer, they will be invited to attend a screening visit if they wish to participate. Participants must sign an NHS REC-approved consent form prior to any study-specific procedures.

### Study treatments

#### Stereotactic radiotherapy

**Fig. 2:**
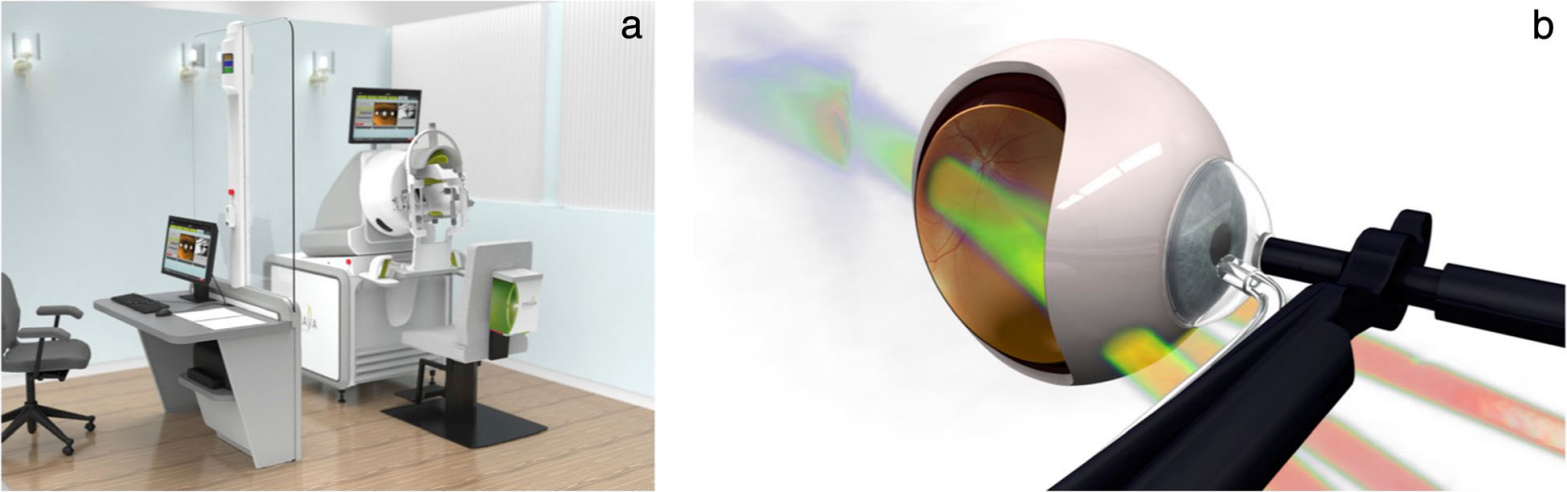
**a** Computer-generated image showing the operator station (*left*) and SRT machine separated by a lead-lined glass window. **b** Image showing the suction-coupled contact lens and position of radiotherapy beams passing through inferior sclera to converge on the macula (images courtesy of Oraya)

SRT will be provided in two or more UK national treatment centres (NTCs). Participants will travel from their recruiting site to the NTC for SRT and then return to their recruiting site for study follow-up. Participants will receive a 16-Gy dose of radiation (or sham treatment) delivered to the macula in a single session using the robotically controlled SRT device, utilising three sequential beams. Each beam deposits 5.3 Gy at the macula, via the pars plana (Fig. 2). If it is not possible to obtain clear access for all three beams, then it may, on occasion, be necessary to deliver radiation through two beams. The dose of radiation will therefore be 8 Gy per beam, identical to the dose delivered in each of the three beams used in the 24 Gy arm of the INTREPID study [16]. Treatment takes about 10–20 minutes.

#### Sham treatment

Participants in the control group will undergo a procedure that is identical to active treatment, but the device will not deliver radiation. The device eye tracking and simulated dose mapping appear identical to those of the live treatment.

#### Ranibizumab treatment

All participants will receive a baseline intravitreal injection of 0.5 mg ranibizumab alongside SRT. Studies indicate that SRT is more effective if given alongside anti-VEGF therapy [11, 19]. Ranibizumab will be administered in the NTC, immediately after SRT. After the initial ranibizumab treatment, participants will be reviewed every 28 days in the recruiting site, and intravitreal 0.5 mg ranibizumab will be administered at that visit if the Comparison of AMD Treatments Trials (CATT) retreatment criteria apply [20], which in summary are:

- The presence of fluid on the OCT (except those eyes in which there has been no decrease in fluid after three consecutive monthly injections)
- Subretinal or intraretinal haemorrhage
- Decreased visual acuity without another explanation
- Increased lesion size or the presence of leakage on fluorescein angiography

### Study assessments

#### Screening (day -14 to day 0)

All ocular assessments will be undertaken on both eyes:

- Demographic information
- Medical and ophthalmic history, including medication use
- Blood pressure
- Best corrected ETDRS VA at 4 m (performed prior to dilating eyes)
- Ophthalmic examination including slit lamp and indirect ophthalmoscopy
- Intraocular pressure (IOP)
- Cataract assessment (Age-Related Eye Disease Study [AREDS] 2008 criteria)
- Optical coherence tomography (OCT, see the following paragraph)
- Fluorescein and indocyanine green angiography (see the following paragraph)
- Fundus photography (see the following paragraph)
- Biometry
- Health-related quality of life and visual function questionnaires

The OCT, fluorescein angiogram and fundus photographs are sent to the independent reading centre at baseline and at months 12, 24, 36 and 48, but not at other visits unless retinopathy is identified. Indocyanine green angiography is only performed at baseline (Table 1).

**Table 1:** Schedule of enrolment, interventions and assessments

Assessment	Screening	SRT with baseline ranibizumab^a^	Monthly review^b^ (months 1–11)	Month 12	Monthly review^b^ (months 13–23)	Month 24	Month 36	Month 48
Visit window: *Day 0 = day of successful enrolment*	Day -14 to 0	Day 0 to 21	±7 days	±7 days	±7 days	±7 days	±14 days	±14 days
Informed consent	X							
Demographics	X							
Ophthalmic history	X							
Med. history/con meds	X							
Blood pressure	X							
ETDRS visual acuity	X		X	X	X	X	X	X
Intraocular pressure	X			X		X	X	X
Cataract assessment	X			X		X	X	X
Biometry	X							
OCT (sent to reading centre)	X			X		X	X	X
OCT (*not* sent to reading centre)	X		X		X			
Fundus photographs (sent to reading centre)	X			X		X	X	X
Fluorescein angiography (sent to reading centre)	X			X		X	X	X
Indocyanine green angiography^c^ (sent to reading centre)	X							
Stereotactic radiotherapy with mandated baseline ranibizumab^a^		X						
Ranibizumab injection if required (*prn*)			X	X	X	X	X	X
Health Economics questionnaires			X	X	X	X	X	X
EQ-5D and VFQ-25 patient questionnaires^d^	X			X		X	X	X
Adverse events/ConMed changes			X	X	X	X	X	X

^a^The baseline mandated ranibizumab injection should be given at the national treatment centres following stereotactic radiotherapy

^b^Monthly review entails review every 28 days rather than by calendar month. The first monthly review should be scheduled 28 ± 7 days after stereotactic radiotherapy/baseline ranibizumab. It is preferable to allow at least 23 days between visits, as this is the minimum time between ranibizumab injections

^c^Indocyanine green may be omitted in centres that do not have indocyanine green capability, if pre-agreed by Sponsor

^d^A treatment satisfaction questionnaire will also be administered by the Sponsor, via telephone. The satisfaction questionnaire is undertaken by central staff, as participants may feel more at liberty to discuss their level of satisfaction without concern that it will affect their local care

Day 0 is defined as the day on which the patient successfully enrols in the study. The measurements recorded during screening constitute the baseline values for subsequent comparison.

#### Stereotactic radiotherapy (day 0 to day 21)

SRT should be administered within 21 days of successful screening (day 0 to 21). SRT and one dose of ranibizumab will be delivered at the NTCs, as described above.

### Monthly review

Participants will return to their recruiting site every 28 days for 24 visits for measurement of ETDRS VA, slit lamp examination of the anterior segment and fundus and OCT, in the study eye. Fluorescein angiography will be undertaken only if clinically indicated. The first monthly review should be 28 days after the initial ranibizumab injection. ETDRS VA and OCT examinations will be undertaken by trial-certified staff, and equipment and ranibizumab will be administered if the CATT retreatment criteria apply [20]. Investigators will record monthly central subfield thickness with manual correction of any segmentation errors.

#### Months 12, 24, 36 and 48

At months 12, 24, 36 and 48 the following will be performed on both eyes:

- ETDRS VA
- Ophthalmic examination
- IOP
- Cataract assessment (AREDS 2008 criteria)
- OCT sent to the reading centre
- Fluorescein angiogram and fundus photographs sent to the reading centre
- Health-related quality of life and visual function questionnaires

The visits at months 36 and 48 are mainly designed to detect any radiation induced-microvascular abnormalities/radiation retinopathy.

### Adverse events and safety reporting

An investigator who detects microvascular abnormalities or signs of radiation retinopathy will forward fundus photographs, angiography and OCT scans to the reading centre. If the reading centre confirms retinopathy or detects a case of retinopathy during routine image review, it will forward the images to a Retinopathy Evaluation Committee. The Retinopathy Evaluation Committee will consist of experts in reading fluorescein angiograms and experts in the clinical characteristics of radiation retinopathy. The Retinopathy Evaluation Committee will decide by majority vote whether or not radiation retinopathy or radiation-related microvascular abnormalities are present. The committee will be the final arbiter as to whether or not radiation retinopathy/microvascular abnormalities are present, but it may review its decision if new, relevant, clinical information emerges for a particular case.

### Sample size calculations

If SRT produces a 25% reduction, group sample sizes of 248 and 124 (ratio: 2:1) achieve 90% power to detect a difference of 2.5 injections between the null hypothesis that both group means are 10 injections and the alternative hypothesis that the mean of the active treatment group is 7.5 injections, with a standard deviation (SD) of 7 for both and a significance level (alpha) of 0.05 (two-sided) using a two-sample *t* test. A 2:1 ratio adds only 42 patients but boosts recruitment and safety data.

We expect VA in the SRT group to be non-inferior compared to the control group. The SD of the mean change in VA was estimated as 12 letters from INTREPID. Group sample sizes of 248 and 124 achieve 97% power to detect non-inferiority in the mean changes in VA using a one-sided, two-sample *t* test assuming an SD of 12 for both groups. The margin of equivalence is 5 letters. The true difference between the means is assumed to be 0. The significance level (alpha) of the test is 0.025.

In the INTREPID study, 2.2% of the randomised population were lost to follow-up by year 1. Year 2 data are not representative, as INTREPID had minimal review in year 2. The CABERNET study had 93% of data available for analysis at the end of year 2. We anticipate a 10% loss to follow-up over 2 years for STAR, so we aim to recruit 274 participants in the active arm and 137 in the control arm (total 411). Sample size calculations were performed using PASS software.

### Justification for parameters used in the sample size calculations

The INTREPID study (ClinicalTrial.gov identifier: NCT01016873) compared patients treated with low-voltage X-ray, external-beam SRT plus ranibizumab *prn* to patients treated with sham SRT plus ranibizumab *prn*. Since INTREPID studied anti-VEGF-experienced patients, the results of that study are more relevant to the STAR population than the results of CATT, which studied anti-VEGF-naïve participants. Participants in INTREPID were randomised to 16 Gy plus ranibizumab *prn*, 24 Gy plus ranibizumab *prn* or sham radiotherapy (either 16 Gy or 24 Gy) plus ranibizumab *prn*. The mean changes in ETDRS VA at 12 months (±SD) were –0.28 ± 8.77, 0.40 ± 10.33 and –1.57 ± 11.90, respectively. The pooled SD across all groups is therefore 10.4, with approximate 95% confidence limits of 9.6 and 11.5. For power calculations for STAR, the assumed SD of the mean change in VA is 12 letters.

The treatment arm of the present study (STAR) will receive 16-Gy SRT, as used in the INTREPID study. Both arms will receive ranibizumab *prn*, as used in the CATT trial. The primary outcome is the ranibizumab re-injection rate over 2 years. CATT reported a mean (±SD) of 6.9 ± 3.0 ranibizumab retreatments to the end of year 1 and 12.6 ± 6.6 to the end of year 2. The year 2 retreatment rate is most relevant to the STAR control group, which recruits patients with previously treated disease (CATT participants were treatment-naive at enrolment). The year 2 CATT retreatment was calculated to be 5.7 injections (12.6 – 6.9), so we might expect our control group to receive twice this amount (11.4) over two years. As CATT was undertaken in the USA, to allow more conservative assumptions in case the injection rate is lower in the UK, we assume the injection rate to be 10 injections over 2 years in our control group, with an SD of 7 (based on INTREPID data which showed the SD was 69% of the mean). A 25% reduction in the number of injections is thought to be clinically and economically meaningful. Notwithstanding the fact that the second year of INTREPID was primarily designed to assess safety and not efficacy, this figure also matches the 25% reduction in the injection rate in the 2 year results of INTREPID, comparing the combined radiotherapy arms to the sham arm (Jackson et al. [17, 18]).

### Proposed timescale

The trial started in December 2014 and is projected to end in October 2022, with a trial duration of 95 months. The duration of each patient’s participation is monthly for 24 months with additional safety visits at months 36 and 48.

### Statistical analyses

In this section we summarise the statistical analysis. Full details are provided in our Statistical Analysis Plan.

Baseline characteristics of each group will be summarised as mean and SD for continuous variables with median and interquartile range for highly skewed data, and count and percentage for categorical variables. No significance testing on baseline variables will be performed.

The baseline characteristics will include patient demographics, randomisation stratifiers, ophthalmic history, medical history, ETDRS visual acuity and other baseline (screening) clinical measures. This will allow an assessment of whether there is clinically important imbalance in any variables.

The main statistical analyses will be conducted according to intention to treat and will estimate the difference in mean outcome between patients randomised to SRT and sham by intention to treat at 24 months. Group difference estimates and associated 95% confidence intervals will be reported. Previous work (CATT and INTREPID) has suggested that the number of injections is approximately normally distributed. In this case, a multiple linear regression analysis will be used to assess the treatment effect with adjustment for the baseline stratification factor (treatment centre). The analysis will not include the initial mandated ranibizumab treatment, as it is administered to all participants and does not reflect the effect of SRT or sham treatment. The treatment effect is evaluated at a two-sided 0.05 significance level. In the event that the number of injections is not normally distributed, a data transformation will be used to give normally distributed residuals. In the unlikely event that no transformation is possible, analysis will be based on a non-parametric approach, a stratified Wilcoxon-Mann-Whitney (WMW) test (the van Elteren test), adjusted for the baseline stratification factors and the median difference with 95% confidence interval calculated by the (stratified) Hodges-Lehmann estimation.

The change in visual acuity (VA) will be formally tested statistically for non-inferiority. The change in VA in the SRT arm compared to the change in VA in the control arm from baseline to month 24 will be analysed by using a multiple linear regression model with adjustment for the baseline stratification factor (treatment centre) and the baseline VA score. Multiple linear regression will be used rather than repeated measure analysis, because although there will be 24 monthly visits for patients in the trial, the focus of interest is the mean changes in VA from baseline to month 24.

Data from the other efficacy outcomes will be summarised. Statistical analysis of these outcomes will be descriptive, with differences and 95% confidence intervals. There will be no correction for multiple testing. Mean vision change and mean OCT thickness will be plotted against time (24 monthly visits over 2 years) as summary measures showing vision change over time and OCT thickness over time.

Subgroup analyses of number of injections, mean VA and OCT thickness (as a forest plot) will be conducted for pre-specified subgroups defined by the following key variables. All subgroup effects will be tested by fitting an interaction factor in the model so that differences between subgroups will only be confirmed if the test for interaction is statistically significant.

1. Total angiographic lesion size, as per reading centre evaluation (above and below the median)
2. Greatest distance of the lesion from the foveal centre, as per the reading centre evaluation
3. Angiographic lesion type per reading centre:
  - Type 1 (occult)
  - Type 2 (classic)
  - Type 3 (retinal angiomatous proliferation [RAP])
  - Mixed (minimally classic)
  - Idiopathic polypoidal choroidal vasculopathy (IPCV)
4. OCT macular volume per reading centre (above and below median)
5. Baseline vision in ETDRS letters (above and below median)
6. Duration of disease (above and below median)
7. Number of prior anti-VEGF injections excluding that given at baseline (above and below median)
8. Presence or absence of vitreomacular adhesion on OCT, as per reading centre
9. Lens status (phakic or pseudophakic)

To address any missingness that occurs, we will conduct a sensitivity analysis of the primary outcome that is adjusted for any factors shown to be different between those present and those with full primary outcome data. The number of patients who have not completed their full treatment protocol is expected to be few, but will be noted. In addition to the primary intention-to-treat analysis, the effect of actually receiving treatment as defined in the protocol will also be estimated by comparing the two arms in just those who have received the full protocol.

Adverse events (AEs), adverse reactions, serious adverse events and serious adverse reactions will be summarised as counts and percentages with 95% confidence intervals by trial arm. Where patients have not received the allocated treatment, this will be noted in reporting AEs so that the denominator for AEs is the number who actually received each treatment.

### Interim analysis

The usual rationale for an interim analysis is to consider stopping the treatment (or the trial). However, as SRT is given at baseline, it is not possible to subsequently stop treatment. As such, we elected not to include an interim analysis. The Data Monitoring Committee will regularly examine the recruitment rate and data completeness and will monitor safety, and the committee will recommend whether the study should continue, stop, be suspended or be modified, based on their findings.

### Economic evaluation

The health economic component of STAR will estimate the relative cost-effectiveness of SRT compared to no SRT and help determine whether SRT provides value for money for the National Health Service. The main outcome measure will be quality of life, which will be used to calculate a cost per QALY gained for SRT plus ranibizumab versus ranibizumab alone.

Participants will complete the National Eye Institute 25 Item Visual Function Questionnaire (VFQ-25) [21] and the EuroQoL EQ-5D [22] at enrolment and then yearly until the study ends at month 48. This provides some indication of the baseline quality of life (in terms of visual function) and a change in response to treatment of the population compared on a common scale with other eye trial adverse populations. The EQ-5D, a generic quality of life questionnaire, will allow comparison of the study results against other (non-vision) health care interventions.

The base case analysis will take an NHS, personal and social services perspective in accordance with National Institute for Health and Care Excellence (NICE) guidance [23, 24]. Since there is no Health Research Group (HRG) code specific to intravitreal injection or AMD monitoring, we will use microcosting estimates of the cost of ranibizumab injections and associated monitoring that were collected previously within the Inhibition of VEGF in Age-related choroidal Neovascularisation (IVAN) trial [25]. This costing work will be replicated to estimate the cost of administering SRT alongside ranibizumab in routine clinical practice. The number of ranibizumab injections, monitoring consultations and ocular imaging procedures (angiography and OCT) will be collected on standard trial forms. At each study visit, participants will be asked to provide data on all eye-related hospital admissions and contacts with medical professionals or eye clinic liaison officers and the reasons for such admissions and contacts, in addition to any residential care, low vision aids and personal care received.

A sensitivity analysis including only costs associated with the study eye or expected adverse events will be conducted. Data on all hospital admissions and outpatient consultations between randomisation and the end of the efficacy study will also be collected from Hospital Episode Statistics to ensure that costs are not underestimated by participant’s recall, missed appointments and/or withdrawal from the study. Analysis of costs and cost-effectiveness will follow standard NICE guidelines [24]. We anticipate using bootstrapping to estimate the uncertainty around incremental costs and QALYs, which will be presented as cost-effectiveness acceptability curves.

### Trial organisation and monitoring

#### Trial Management Committee

The Trial Management Committee consists of:

Mr Timothy Jackson, Chief Investigator, Consultant Ophthalmic Surgeon, King’s College London, London, UK

Mrs Riti Desai, Clinical Trials Manager, King’s College Hospital, London, UK

Ms Joanna Kelly, Strategic Data Management Lead, King’s Clinical Trials Unit, King’s College London, UK

Ms Caroline Murphy, Operational Director, King’s Clinical Trials Unit, King’s College London, UK

Dr Yanzhong Wang, Senior Lecturer in Medical Statistics, King’s College London, UK

Ms Beverley White-Alao, Trial Management Strategic Lead, King’s Clinical Trials Unit, King’s College London, UK

#### Trial Steering Committee

The Trial Steering Committee consists of:

Mr Richard Wormald, Cochrane Eyes and Vision Group, International Centre for Eye Health, London School of Hygiene and Tropical Medicine, London, UK (independent voting clinical Chair)

Prof. Winfried Amoaku, Associate Professor and Reader in Ophthalmology and Visual Sciences, University of Nottingham, UK (independent voting clinician)

Ms Clare Bailey, Consultant Ophthalmic Surgeon, Bristol Eye Hospital, Bristol, UK (independent voting clinician)

Mr Timothy Jackson, Consultant Ophthalmic Surgeon, King’s College London, London, UK (voting clinical Chief Investigator)

Mr Luke Membrey, Consultant Ophthalmic Surgeon, Maidstone Hospital, Kent, UK (non-voting Principal Investigators’ representative)

Mr Barnaby Reeves, Professor of Health Services Research, University of Bristol, Bristol, UK (non-voting trialist)

Mr Mandeep Sagoo, Consultant Ocular Oncologist, St Bartholomew’s Hospital, London, UK (independent voting clinician)

Dr Yanzhong Wang, Senior Lecturer in Medical Statistics, King’s College London, London, UK (voting Trial Statistician)

Prof. Robert West, Professor of Biostatistics, Leeds Institute of Health Sciences, Leeds, UK (independent voting statistician)

Ms Cathy Yelf, Head of External Relations, Macular Society, London, UK (non-voting Lay Representative)

#### Data Monitoring Committee

The Data Monitoring Committee consists of:

Prof. Craig Ramsay, Statistician, Health Services Research Unit, University of Aberdeen, UK

Prof. Paulo Stanga, Consultant Ophthalmologist and Vitreoretinal Surgeon, Manchester Royal Eye Hospital, Manchester, UK

Prof. Heinrich Heimann, Consultant Ocular Oncologist, Royal Liverpool University Hospital, Liverpool, UK

## Key protocol amendments

The definition of the minimum OCT macular volume required for inclusion changed from the 95^th^ percentile of normal to defined values for each of the machines in use across sites. Following amendment, the minimum macular volume for the Heidelberg Spectralis machine was 8.15 mm^3^, Topcon 3D-OCT 7.53 mm^3^, Optovue 6.14 mm^3^, and Zeiss Cirrus 10.3 mm^3^. A minimum macular volume formed part of the eligibility criteria, as a subgroup analysis of the INTREPID study found macular volume to be a key driver of outcome [18]. However, INTREPID used an older, time-domain, OCT machine (Stratus, Carl Zeiss Meditec, Cambridge, UK). The conversion between Stratus OCT and the new SD-OCT machines used in STAR was initially handled via each machine’s normative database or published values, but the amendment reduced the macular volume threshold to one we believe to more closely match the INTREPID subgroup threshold, based on discussions with machine manufacturers and our own data collection in patients with wet AMD.

Initially, patients likely to require cataract surgery within 2 years of enrolment were excluded, but this exclusion criterion was removed, as the emerging literature suggested that stereotactic radiotherapy does not cause cataracts. We relaxed the requirement that participants need to have had an anti-VEGF injection within 3 months of enrolment to 4 months, to facilitate recruitment of an increasing proportion of patients receiving aflibercept, who typically attend 2 monthly rather than monthly. We made small edits to clarify the measurement of lesion size and distance of the lesion to the fovea. Finally, to expand the number of sites, we removed the requirement to undertake a baseline indocyanine green (ICG) angiography for sites without ICG capability.

## Discussion

AMD is the leading cause of blindness in developed nations, and the incidence is projected to increase as the population ages [26]. Wet AMD is treated with repeated intravitreal anti-VEGF injections from the point of diagnosis. Whilst these injections have a favourable safety profile, with visual outcomes far better than the natural history, the treatment is burdensome and expensive, and is associated with small but repeated risks of injection-related complications.

A randomised, double-masked, sham-controlled phase II study suggests that SRT may reduce the burden of nAMD treatment by significantly reducing the number of injections that patients require [16, 17]. STAR is a phase III randomised, sham-controlled, double-masked clinical trial that evaluates the safety and efficacy of SRT. It targets those patients thought to be most responsive to SRT, to test the hypothesis that SRT reduces the frequency of ranibizumab injections. A 2:1 ratio was selected to encourage enrolment, on the assumption that many of those wishing to join the trial did so hoping to receive the new treatment. It will also provide long-term safety data using specialised imaging to look for collateral damage from radiation, which previous studies suggest can be subtle and with delayed onset.

The risk of bias is thought to be low, as the SRT device produces very effective masking for the participant and operator, such that all subsequent observations are concealed to treatment allocation. One challenge of the trial is that radiation damage can be very subtle, and sometimes relatively non-specific. In the INTREPID study of SRT, only two cases were initially detected by examining clinicians, and most were instead detected by a reading centre, using specialised imaging (fundus photographs with fluorescein angiography). Although most cases of microvascular abnormality occurred outside the fovea and therefore did not affect vision, some did involve the fovea and it can be difficult to determine if any loss of VA is due to radiation or the underlying nAMD process. To deal with this uncertainty, an independent expert committee will adjudicate cases, but it is recognised that a definitive conclusion may not be reached in all cases of suspected microvascular abnormality.

If STAR demonstrates that SRT is safe and effective, then it has the potential to change the treatment landscape and reduce the burden of treatment faced by the growing number of people with wet AMD.

## Trial status

At the time of manuscript submission, the recruitment to the STAR trial is ongoing.

## Abbreviations

AE: Adverse event
AMD: Age-related macular degeneration
CNV: Choroidal neovascularisation
CRF: Case report form
CTIMP: Clinical Trial of an Investigational Medicinal Product
EMB: Epimacular brachytherapy
EME: Efficacy and Mechanism Evaluation Programme
ETDRS: Early Treatment of Diabetic Retinopathy Study
FA: Fluorescein angiography
ICD: Implantable cardioverter defibrillator
ICG: Indocyanine green
IOP: Intraocular pressure
IPCV: Idiopathic polypoidal choroidal vasculopathy
LASIK: Laser-assisted in situ keratomileusis
MHRA: Medicines and Healthcare products Regulatory Agency
nAMD: Neovascular age-related macular degeneration
NICE: National Institute for Health and Care Excellence
NIHR: National Institute for Health Research
NTC: National treatment centre
OCT: Optical coherence tomography
PDT: Photodynamic therapy
POH: Presumed ocular histoplasmosis
*prn*: *pro re nata*
QALY: Quality-adjusted life year
RAP: Retinal angiomatous proliferation
SAE: Serious adverse event
SAP: Statistical Analysis Plan
SD: Standard deviation
SRT: Stereotactic radiotherapy
TTT: Transpupillary thermotherapy
VA: Visual acuity
VEGF: Vascular endothelial growth factor

## References

[1] Klein R, Klein BE, Jensen SC, Mares-Perlman JA, Cruickshanks KJ, Palta M (1999). Age-related maculopathy in a multiracial United States population: the National Health and Nutritional Examination Survey III. Ophthalmology.

[2] Owen CG, Jarrar Z, Wormald R (2012). The estimated prevalence and incidence of late stage age related macular degeneration in the UK. Br J Ophthalmol.

[3] Brown DM, Kaiser PK, Michels M (2006). Ranibizumab versus verteporfin for neovascular age-related macular degeneration. N Engl J Med.

[4] Rosenfeld PJ, Brown DM, Heier JS (2006). Ranibizumab for neovascular age-related macular degeneration. N Engl J Med.

[5] Amoaku WM, Chakravarthy U, Gale R (2015). Defining response to anti-VEGF therapies in neovascular AMD. Eye (Lond).

[6] Miyamoto H, Kimura H, Yasukawa T (1999). Effect of focal X-ray irradiation on experimental choroidal neovascularization. Invest Ophthalmol Vis Sci.

[7] Chakravarthy U, Gardiner TA, Archer DB, Maguire CJ (1989). A light microscopic and autoradiographic study of non-irradiated and irradiated ocular wounds. Curr Eye Res.

[8] Aisenbrey S, Lafaut BA, Reynders S (2003). Clinicopathological correlation of choroidal neovascularization after external beam radiotherapy in age-related macular degeneration. Graefes Arch Clin Exp Ophthalmol.

[9] Canton VM, Quiroz-Mercado H, Velez-Montoya R (2011). 16 Gy low-voltage x-ray irradiation with ranibizumab therapy for AMD: 6-month safety and functional outcomes. Ophthalmic Surg Lasers Imaging.

[10] Canton VM, Quiroz-Mercado H, Velez-Montoya R (2012). 24-Gy low-voltage x-ray irradiation with ranibizumab therapy for neovascular AMD: 6-month safety and functional outcomes. Ophthalmic Surg Lasers Imaging.

[11] Moshfeghi AA, Morales-Canton V, Quiroz-Mercado H (2012). 16 Gy low-voltage x-ray irradiation followed by as needed ranibizumab therapy for age-related macular degeneration: 12 month outcomes of a ‘radiation-first’ strategy. Br J Ophthalmol.

[12] Petrarca R, Jackson TL (2011). Radiation therapy for neovascular age-related macular degeneration. Clin Ophthalmol.

[13] Neffendorf JE, Jackson TL (2015). Stereotactic radiotherapy for wet age-related macular degeneration: current perspectives. Clin Ophthalmol.

[14] Moshfeghi DM, Kaiser PK, Gertner M (2011). Stereotactic low-voltage x-ray irradiation for age-related macular degeneration. Br J Ophthalmol.

[15] Hanlon J, Firpo M, Chell E (2011). Stereotactic radiosurgery for AMD: a Monte-Carlo-based assessment of patient-specific tissue doses. Invest Ophthalmol Vis Sci.

[16] Jackson TL, Chakravarthy U, Kaiser PK (2013). Stereotactic radiotherapy for neovascular age-related macular degeneration: 52-week safety and efficacy results of the INTREPID study. Ophthalmology.

[17] Jackson TL, Chakravarthy U, Slakter JS (2015). Stereotactic radiotherapy for neovascular age-related macular degeneration: year 2 results of the INTREPID study. Ophthalmology.

[18] Jackson TL, Shusterman EM, Arnoldussen M, Chell E, Wang K, Moshfeghi DM (2015). Stereotactic radiotherapy for wet age-related macular degeneration (INTREPID): influence of baseline characteristics on clinical response. Retina.

[19] Morales-Canton V, Quiroz-Mercado H, Velez-Montoya R (2013). 16 and 24 Gy Low-voltage x-ray irradiation with ranibizumab therapy for neovascular age-related macular degeneration: 12-month outcomes. Am J Ophthalmol.

[20] Group CATT, Martin DF, Maguire MG (2011). Ranibizumab and bevacizumab for neovascular age-related macular degeneration. N Engl J Med.

[21] Mangione CM, Lee PP, Gutierrez PR (2001). Development of the 25-item National Eye Institute Visual Function Questionnaire. Arch Ophthalmol.

[22] The EuroQoL Group (1990). EuroQoL-a new facility for the measurement of health-related quality of life. Health Policy.

[23] Drummond MF, Sulpher MJ, Torrance GW (2005). Methods for the economic evaluation of health care programmes.

[24] NICE. Guide to the methods of technology appraisal 2013. London: National Institute of Health and Care Excellence; 2013. 27905712

[25] Investigators IS, Chakravarthy U, Harding SP, et al. Ranibizumab versus bevacizumab to treat neovascular age-related macular degeneration: one-year findings from the IVAN randomized trial. Ophthalmology 2012;119(7):1399-411. 10.1016/j.ophtha.2012.04.015 22578446

[26] Velez-Montoya R, Oliver SCN, Olson JL, Fine SL, Quiroz-Mercado H (2014). Mandava N. Current knowledge and trends in age-related macular degeneration.

